# Supplementary material for: Reconciling Mining with the Conservation of Cave Biodiversity: A Quantitative Baseline to Help Establish Conservation Priorities
Source: PLoS One. 2016 Dec 20;11(12):e0168348. doi: 10.1371/journal.pone.0168348 (PMC5173368; doi:10.1371/journal.pone.0168348)
Supplement: S1 Dataset — (ZIP) [file pone.0168348.s002.zip › Taxa/Serra Norte/SN_2013/N4WS/Lista N4WS-62.pdf]

CAVIDADE N4WS-0062

Classe	Ordem	Fam/Outros	Gên/Outros	Espécie	S-U	U-S	Zon.
Arachnida	Acari	(Oribatida)		sp.2		X	E
Arachnida	Acari	(Parasitengonina)		sp.1		X	E
Arachnida	Acari	Argasidae	*Ornithodoros*	sp.1	X		P
Arachnida	Acari	Ixodidae	*Amblyomma*	sp.1	X		E
Arachnida	Amblypygi	Charinidae	*Charinus*	sp.1		1	E
Arachnida	Araneae	Araneidae	*Alpaida*	*septemmammata*		X	E
Arachnida	Araneae	Caponiidae		jovem		X	E
Arachnida	Araneae	Ctenidae		jovem		1	E
Arachnida	Araneae	Drymusidae		jovem	X	X	E/P
Arachnida	Araneae	Hahniidae		jovem		X	E
Arachnida	Araneae	Ochyroceratidae	*Ochyrocera*	sp.1	X	X	E
Arachnida	Araneae	Pholcidae	*Leptopholcus*	sp.1		X	E
Arachnida	Araneae	Pholcidae	*Mesabolivar*	*eberhard*	X	X	E
Arachnida	Araneae	Prodidomidae		jovem		X	E
Arachnida	Araneae	Scytodidae	*Scytodes*	*globula*	2	8	E/P
Arachnida	Araneae	Theraphosidae		jovem		3	P
Arachnida	Araneae	Theridiidae	*Dipoena*	sp.1	X		E
Arachnida	Araneae	Uloboridae	*Miagrammopes*	sp.1	X		E
Arachnida	Araneae	Uloboridae	*Uloborus*	sp.1	X	X	E
Arachnida	Opiliones	Escadabiidae		sp.1	X		P
Arachnida	Opiliones	Sclerosomatidae	*Prionostema*	sp.1		X	E
Arachnida	Pseudoscorpiones	Chthoniidae	*Pseudochthonius*	sp.1		X	E
Chilopoda	Scolopendromorpha	Scolopocryptopidae	*Newportia*	sp.1		1	E
Diplopoda	Spirostreptida	Pseudonannolenidae	*Pseudonannolene*	sp.1		2	E
Entognatha	Collembola	Entomobryidae		sp.8		X	E
Entognatha	Collembola	Paronellidae		sp.1	X	X	E/P
Entognatha	Collembola	Paronellidae		sp.4		X	E
Entognatha	Diplura	Campodeidae		sp.1		X	E
Insecta	Blattodea			jovem	2	1	E/P
Insecta	Coleoptera	Dytiscidae		sp.12A		X	E
Insecta	Diptera	Cecidomyiidae	Cecidomyiinae	sp.	X	X	E
Insecta	Diptera	Culicidae	Sabethini	sp.	X		E
Insecta	Diptera	Dolichopodidae		sp.	X		E
Insecta	Diptera	Psychodidae	*Sciopemyia*	*sordellii*	X	X	E/P
Insecta	Diptera	Tipulidae	Tipulinae	sp.	X		P
Insecta	Heteroptera	Lygaeidae	Rhyparochrominae	sp.1		X	E
Insecta	Heteroptera	Reduviidae	Emesinae	jovem		3	E
Insecta	Homoptera	Cixiidae		sp.1		X	E
Insecta	Homoptera	Cixiidae		sp.3	X		E
Insecta	Hymenoptera	Formicidae	*Camponotus*	sp.1	X	X	E
Insecta	Hymenoptera	Formicidae	*Dolichoderus*	aff. *bispinosus*	X		E
Insecta	Hymenoptera	Formicidae	*Pachycondyla*	*striata*	X		P
Insecta	Hymenoptera	Formicidae	*Wasmannia*	sp.1		X	E
Insecta	Isoptera	Termitidae	*Embiratermes*	sp.		X	E
Insecta	Isoptera	Termitidae	*Nasutitermes*	sp.		X	E
Insecta	Isoptera	Termitidae	*Spinitermes*	sp.		X	P
Insecta	Isoptera			sp.	X		P
Insecta	Lepidoptera	Noctuoidea		sp.1	2		E
Insecta	Lepidoptera	Noctuoidea		sp.12	1		E

Insecta	Neuroptera	Mantispidae	*Plega*	sp.	1		E
Insecta	Orthoptera	Phalangopsidae	*Paraclodes*	sp.	10	4	E
Insecta	Orthoptera	Phalangopsidae	*Phalangopsis*	sp.	1	1	P
Insecta	Psocoptera	Psocomorpha		jovem	X	X	E
Malacostraca	Isopoda	Armadillidae		sp.1	X		P
Malacostraca	Isopoda	Dubioniscidae		sp.1		X	E
Amphibia	Anura	Strabomantidae	*Pristimantis*	cf. *fenestratus*	5	2	E/P
Reptilia	Testudinata	Testudinidae	*Chelonoidis*	sp.		1	E
